# Schmallenberg virus epidemiology and regional control strategies: diagnostics, vaccines, and vector management

**DOI:** 10.3389/fcimb.2025.1633030

**Published:** 2025-07-14

**Authors:** Jing Wang, Qi Jia, Haoyu Xiang, Fang Wang, Chao Sun, Jitao Chang, Zhigang Jiang, Xin Yin

**Affiliations:** ^1^ State Key Laboratory for Animal Disease Control and Prevention, Harbin Veterinary Research Institute, Chinese Academy of Agricultural Sciences, Harbin, China; ^2^ TERRA Teaching and Research Center, Gembloux Agro-Bio Tech, University of Liège, Liège, Belgium; ^3^ College of Animal Science and Technology, Ningxia University, Yinchuan, China

**Keywords:** Schmallenberg virus, diagnostic approaches, vaccination strategies, vector control, endemic regions, non-endemic regions

## Abstract

Schmallenberg virus (SBV) is an emerging orthobunyavirus transmitted by *Culicoides* midges. It poses a serious global health threat to ruminants, especially during pregnancy, causing abortion, stillbirths, and congenital malformations. Since its first outbreak in 2011, SBV has spread across Europe and other regions. Its transmission has expanded due to global climate change and increased animal trade, resulting in recurrent outbreaks in endemic regions and a growing risk of introduction into non-endemic areas. This situation highlights the urgent need for improved control strategies. This review summarizes the pathogenic and epidemiological characteristics of SBV and provides an overview of recent advancements in diagnostic approaches, vaccine development, and vector control. Diagnostic approaches, such as serological assays and nucleic acid-based tests, have become the primary tools for SBV detection. However, their applicability in clinical settings still requires further optimization. In terms of vaccine development, existing inactivated vaccines have limitations, including the inability to distinguish between vaccinated and infected animals. This has driven the development of next-generation vaccines, such as recombinant protein, viral vector, and mRNA-based platforms. For vector control, integrated approaches combining chemical, ecological, and biological strategies have been proposed to interrupt the transmission of the virus by *Culicoides* midges. Additionally, this review emphasizes the necessity of region-specific control strategies tailored to the differing epidemiological contexts. In endemic regions, comprehensive measures, including pathogen surveillance, vaccination programs, and *Culicoides* control, are critical. In non-endemic regions, the focus should be on enhancing border biosecurity, monitoring international trade, and establishing early warning systems. These strategies not only provide a scientific foundation for SBV control but also offer practical guidance for managing the spread of similar vector-borne viruses globally.

## 1 Introduction

Schmallenberg virus (SBV) was first identified in November 2011 in plasma samples from dairy cows suffering from fever and diarrhea near the town of Schmallenberg, Germany (Hoffmann et al., 2012). The virus was subsequently named after this location. SBV belongs to the Simbu serogroup within the *Orthobunyavirus* genus and shares a high degree of homology with Akabane virus (Sick et al., 2019). Genomic analysis has revealed frequent recombination events in the S, M, and L genome segments of SBV, which help it adapt to to new hosts and environments (Hughes et al., 2020). The virus is primarily transmitted by *Culicoides* and is associated with significant reproductive failures in ruminants, including abortion, stillbirth, and arthrogryposis-hydranencephaly syndrome (AHS) (Sibhat et al., 2018). Within a few months, SBV spread across Western Europe, causing significant economic losses to the livestock industry (Stavrou et al., 2017). According to EU statistics, direct losses to the livestock sector exceeded €150 million at the peak of the outbreak in 2012 (Charlier et al., 2020). In recent years, epidemiological data indicate that the virus has spread beyond Europe (Bradshaw et al., 2012; Mason et al., 2013; Rasmussen et al., 2014; Barrett et al., 2015). Combined with the seasonal expansion of *Culicoides* vectors, this has created a complex “host-vector-environment” transmission network.

The current prevention and control system has three main challenges. First, existing diagnostic methods like pathogen-based, nucleic acid, and serological tests cannot provide rapid on-site detection (Mansfield et al., 2013; Wernike and Beer, 2019). Additionally, serological testing has a high false-positive rate due to cross-reactivity with other Simbu serogroup viruses, such as Akabane virus (Bilk et al., 2012; Loeffen et al., 2012; Bréard et al., 2013). Second, although inactivated vaccines against SBV have demonstrated efficacy, their use remains limited due to the requirement for multiple doses and lack of DIVA capability. Moreover, current research progress on the duration of immunity remains insufficient and needs further investigation. Furthermore, the unpredictable and seasonal circulation of SBV reduces the willingness of farmers to vaccinate, resulting in poor uptake and, in some regions, withdrawal of the vaccine from the market (Wernike and Beer, 2020). Third, traditional vector control strategies are losing effectiveness due to the evolution of insecticide resistance in *Culicoides* midges (Rasmussen et al., 2014; Naqqash et al., 2016; Sick et al., 2019). Furthermore, studies on the virus’s overwintering mechanisms have indicated that in regions above 45°N latitude, *Culicoides* midges undergo diapause for up to five months (Purse et al., 2005), yet the virus can still maintain its ecological niche through vertical transmission via the placenta (De Regge et al., 2012; Wernike et al., 2013c; Poskin et al., 2017). This poses a risk for cross-border spread. Moreover, since SBV infections in adult, non-pregnant ruminants are typically asymptomatic or very mild, monitoring efforts are further complicated (Afonso et al., 2014; Wernike et al., 2014). The traditional “one-size-fits-all” approach is no longer sufficient to address the ecological complexity of the virus. There is an urgent need to develop a precise prevention and control system based on geographically stratified transmission risk assessments.

This article provides a comprehensive review of the diagnostic approaches, vaccine development, and vector control strategies for SBV. Additionally, it introduces an innovative “epidemic regions vs. non-epidemic regions” dual-track prevention and control strategy. Based on regional differences, this article proposes scientifically sound and practical control measures aimed at enhancing prevention effectiveness. The goal is to provide theoretical foundations and technical support for global epidemic control efforts.

## 2 SBV classification and genome

**Figure 1:**
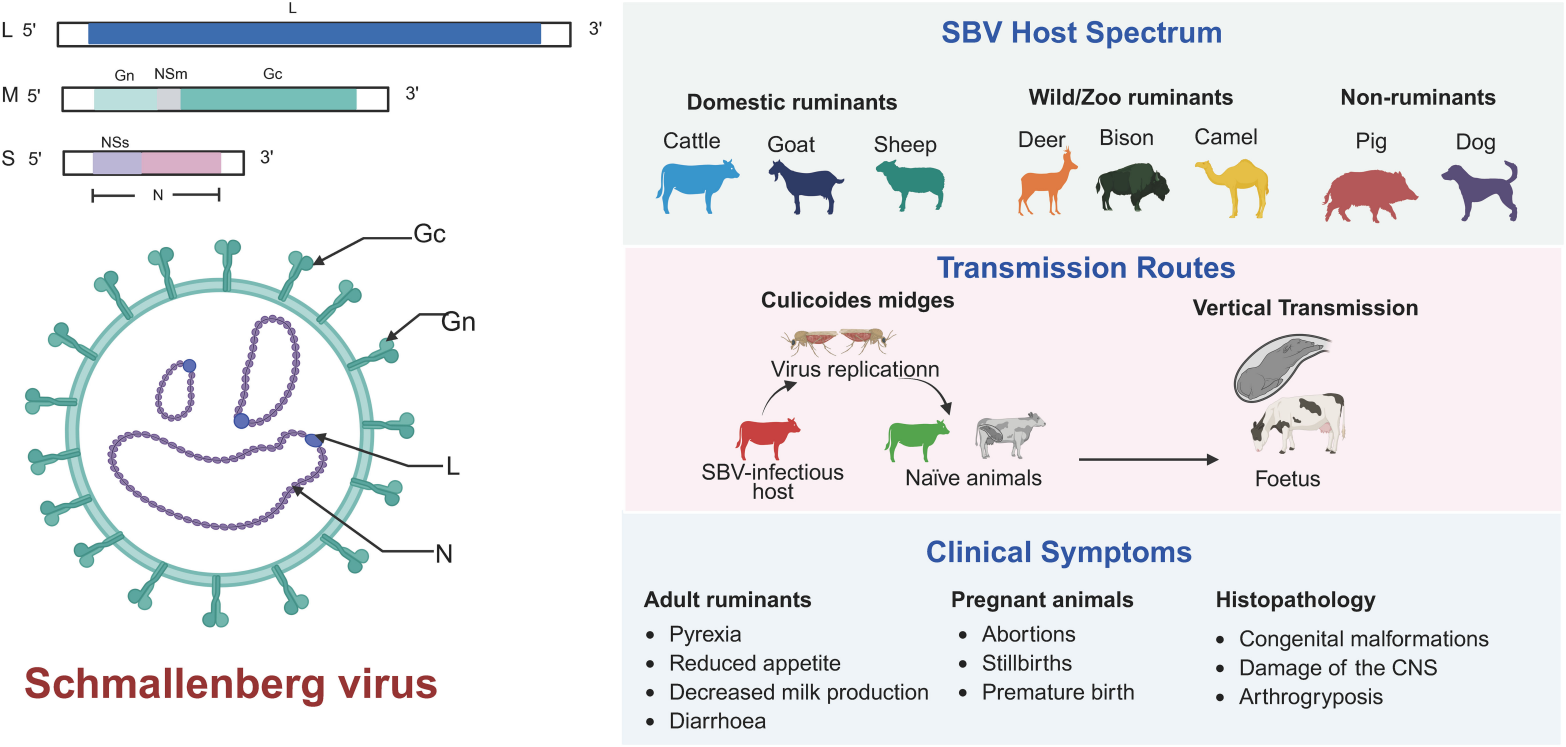
Overview of SBV structure, host spectrum, transmission routes, and clinical symptoms.

SBV is a Simbu serogroup virus belonging to the genus *Orthobunyavirus* within the family *Peribunyaviridae* (Saeed et al., 2001; Rasekh et al., 2018). The genus *Orthobunyaviridae* contains over 170 species of viruses, including those that cause human diseases (such as Oropouche virus and La Crosse virus) and ruminant diseases (such as Akabane virus, Aino virus, Cache Valley Fever virus) (Abudurexiti et al., 2019). The genome of SBV consists of three negative-strand RNA segments: L segment (large), M segment (medium) and S segment (small) (**Figure 1**) (Bouloy et al., 1973). The L segment encodes the RNA-dependent RNA polymerase (RdRp) of the virus, which is responsible for viral replication and transcription (Walter and Barr, 2011). The Gn and Gc glycoproteins are essential components of the viral envelope, mediating viral adsorption and membrane fusion (Wernike et al., 2018b). Both glycoproteins serve as the primary targets of the host immune system and can elicit specific antibody responses (Endalew et al., 2019; Wang et al., 2024). Notably, Gc demonstrates particularly strong immunogenicity and has been identified as the predominant target for the production of potent neutralizing antibodies (Wang et al., 2024). Extensive characterization of their antigenic epitopes, especially those on Gc, provides crucial molecular insights for vaccine design, offering strategies to enhance immunogenicity and protective efficacy. The S segment encodes the nucleocapsid protein (N) and the non-structural protein NSs. The N protein is a core component of the viral replication complex, encapsidating viral RNA to form a ribonucleoprotein (RNP) complex, which protects the genome and facilitates viral replication (Ak et al., 2007; Ariza et al., 2013). *In vitro* studies have shown that deletion of NSs impairs viral replication in interferon-sensitive cells, prevents interferon (IFN) synthesis suppression in infected cells, and disrupts host protein synthesis shutdown (Varela et al., 2016). NSs has been identified as a major virulence factor that downregulates host mRNA synthesis and type I IFN production in mammalian cells, thus enhancing viral replication (Thomas et al., 2004).

The segmented genome characteristic of SBV not only enable the virus to efficiently adapt to different hosts and environments but also significantly enhance its genetic diversity through recombination and reassortment. This provides an evolutionary advantage for the virus in terms of host adaptation (Yanase et al., 2003, 2010; Varela et al., 2016). Due to the segmented genome, the virus can flexibly respond to environmental pressures, such as host immune responses or climate changes, maintaining its infectivity through rapid evolution (Wernike et al., 2021; Sick et al., 2024). Additionally, the segmented genome may facilitate genetic exchange with other *Orthobunyavirus* species. In the event of co-infection within a host, new recombinant viruses could potentially emerge. This potential genetic compatibility not only influences the ecological adaptability of the virus but also pose public health concerns. Therefore, continuous monitoring of the genetic evolution of SBV, especially interspecies reassortment, is crucial for preventing the emergence of novel pathogens and for informing effective epidemiological surveillance and control strategies. Notably, virus variants isolated from malformed fetuses frequently carry specific mutations, particularly in the S and M genome segments, which impair replication in insect cells and indicate a loss of fitness for vector transmission. Such variants likely do not participate in the natural transmission cycle between mammalian hosts and insect vectors. Studying these mutations is essential to understanding the protein functions that are critical for viral adaptation to different host (Sick et al., 2024).

## 3 SBV epidemiological characteristics

**Figure 2:**
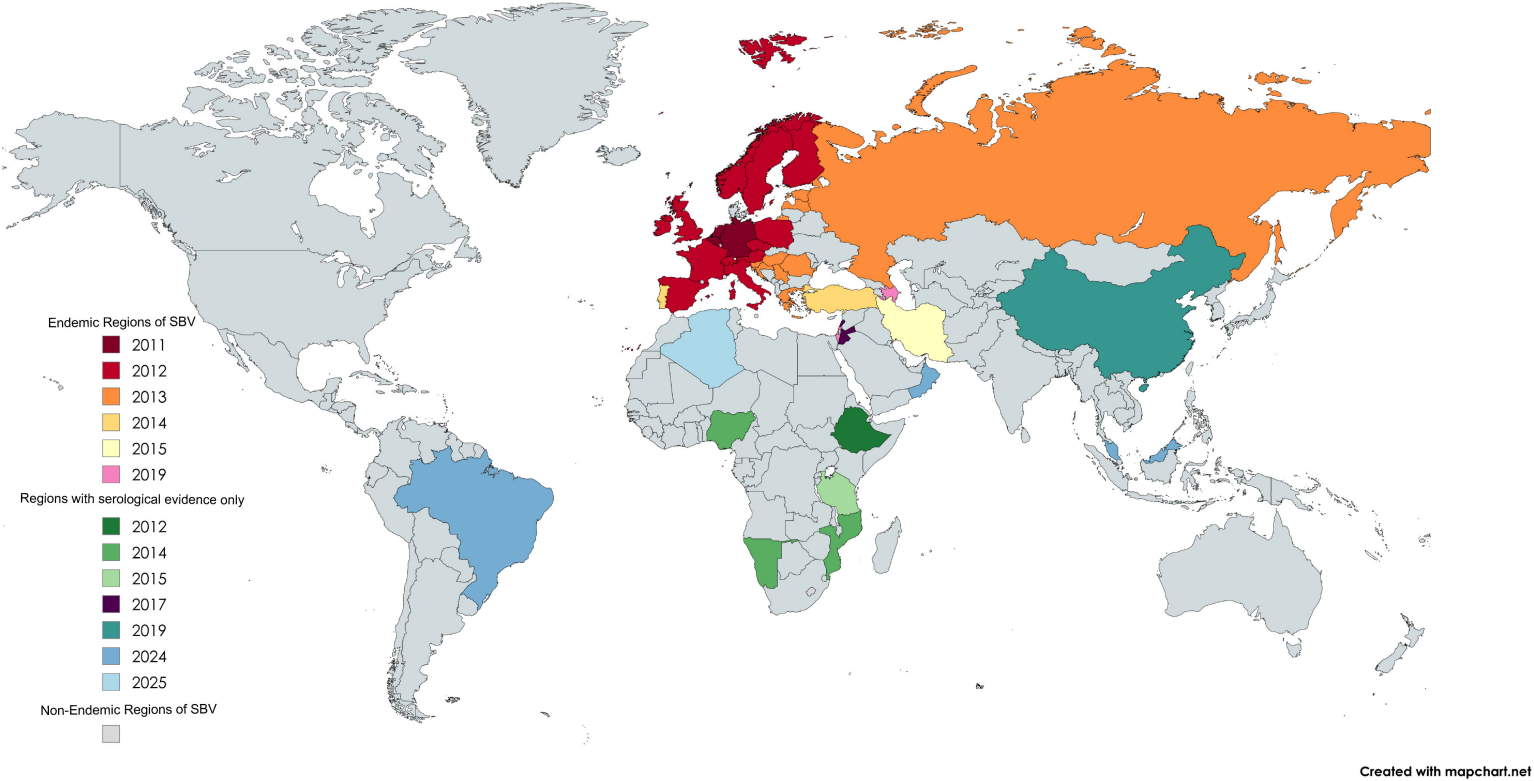
SBV distribution by country and year of first reported case. Map generated using MapChart.net.

Since its first outbreak in Northern Europe in the autumn of 2011, SBV has exhibited a clear trend of cross-border transmission (**Figure 2**). In 2012, the virus spread rapidly from the British Isles to Scotland and Ireland (Conraths et al., 2013; Dominguez et al., 2014), further expanding into Eastern Europe and the Mediterranean region. Within a year, SBV had spread across Europe and evolved into an endemic pathogen with periodic outbreaks occurring every two to three years. For instance, outbreaks re-emerged in Ireland, the United Kingdom, and Belgium during 2016–2017 (Stokes et al., 2016; Collins et al., 2017; Veldhuis et al., 2017). Notably, in 2022, Germany reported a seroprevalence of 4.92%, which surged to 40.15% in 2023, indicating a significantly rise in viral activity (Wernike et al., 2022). Juvenile ruminants exhibited a seroprevalence of 31.82% in 2023, while the overall adult seroprevalence reached 40.15%, signaling a large-scale outbreak during the summer and autumn months. Although Europe remains the primary endemic region, SBV has expanded to other continents, including Africa and Asia. SBV circulation has been confirmed in Turkey, where it was first detected in 2014 through molecular analysis of aborted ruminant fetuses and showed 29.11% seropositivity and 3.17% PCR positivity in ruminants between 2015 and 2017 in the Eastern Mediterranean region, as well as in Israel in 2019, where genomic detection in both *Culicoides* midges and affected ruminants demonstrated its presence (Yilmaz et al., 2014; Abutarbush et al., 2017; Behar et al., 2021). Additionally, several countries have also reported serological evidence of SBV, although no pathogen-based evidence has been obtained so far. For example, in 2018, Ethiopia reported seroprevalence rates of 56.6% at the individual level and 82.9% at the herd-level (Sibhat et al., 2018). Meanwhile, serological evidence confirmed its presence in East Asia, specifically in Guangdong Province, China (Zhai et al., 2018). A 2023 study first reported serological evidence of SBV infection in large populations of sheep and goats across multiple states in Peninsular Malaysia. The high seroprevalence of up to 27.8% indicates significant viral circulation among local small ruminants (Jimale et al., 2024). Furthermore, a recent global meta-analysis indicates infection rates of 49% in domestic ruminants and 26% in wild ruminants (Dagnaw et al., 2024).

SBV is primarily transmitted by the bites of haematophagous midges of the genus *Culicoides*. These arthropods serve as the principal biological vectors of the virus (Sick et al., 2019). Transmission occurs when an uninfected midge takes a blood meal from an SBV-infected ruminant—typically cattle, sheep, or goats. During the extrinsic incubation period (EIP), the virus replicates within the midge until it reaches transmissible levels. The infected midge can then pass the virus to another host during subsequent feeding. Several *Culicoides* species have been implicated in SBV transmission, particularly those in the Obsoletus group (Larska et al., 2013). Additionally, vertical (transovarial) transmission within the vector population has been proposed as a mechanism for viral persistence. This is supported by the detection of SBV RNA in nulliparous *Culicoides*—unfed midges—suggesting that the virus may be transmitted from adult females to their offspring. Such vertical transmission could enable SBV to overwinter within vector populations, thereby facilitating viral re-emergence during subsequent transmission seasons.

SBV is also capable of vertical transmission in ruminant hosts. When a pregnant animal becomes infected, viremia may allow the virus to cross the placenta, infecting the developing fetus (Herder et al., 2012). If infection occurs during critical gestational windows, it can result in congenital malformations, stillbirths, or abortions. Although the exact susceptible period remains undefined, it is estimated to be between gestational days 28–56 in small ruminants and days 80–150 in cattle (Hartley et al., 1977; Parsonson et al., 1988). Detection of SBV RNA in malformed neonates and aborted fetuses has confirmed this route of transmission in field settings (Dogan et al., 2022).

Direct horizontal transmission between animals appears to be unlikely. While experimental subcutaneous inoculation of cattle has resulted in SBV RNA detection in fecal, oral, and nasal swabs, oral or nasal inoculation did not lead to productive infection or seroconversion (Wernike et al., 2013a). These findings indicate that direct transmission through these routes under natural conditions is improbable.

The clinical symptoms of SBV infection vary depending on the host species and age. SBV primarily affects domestic ruminants such as cattle, sheep, and goats, but it has also been detected in wild ruminants, including roe deer and bison (Lievaart-Peterson et al., 2015a; Jiménez-Ruiz et al., 2021, 2022). However, clinical symptoms associated with SBV infection in wild ruminants have not been reported, and further research is needed to elucidate the impact of SBV infection on these species and their role in SBV epidemiology (Schulz et al., 2015; García-Bocanegra et al., 2017). In adult cattle, SBV infection is often asymptomatic or mild, with common symptoms including transient fever, reduced appetite, and decreased milk production, generally resolving within a few days (Wernike et al., 2012). In contrast, adult sheep and goats mainly experience subclinical infections, with only a few acute cases presenting symptoms such as fever, diarrhea, or reduced milk yield (Afonso et al., 2014). However, in pregnant animals, the virus can cross the placenta and infect the fetus, leading to abortion, premature birth, stillbirth, or congenital malformations such as arthrogryposis, hydrocephalus, and porencephaly (De Regge, 2017; Endalew et al., 2018). SBV infection in pregnant animals differ according to the gestational stage at the time of infection. Infection during early pregnancy typically results in embryo loss or abortion, while infection in mid-gestation often causes severe fetal malformations, particularly affecting the central nervous system and musculoskeletal development. Infections occurring late in pregnancy may lead to inflammatory lesions in the fetal brain and neurological symptoms in neonates (O’Connor et al., 2024). There is an inverse correlation between herd immunity levels and the incidence of congenital malformations—higher herd immunity leads to fewer new infections and a reduced rate of congenital defects. Conversely, when the number of susceptible individuals increases, the risk of infection in pregnant animals rises, leading to a higher incidence of fetal malformations. In endemic regions where SBV has circulated for years, most adult animals have acquired natural immunity through prior infection or vaccination. As a result, new infections are relatively rare, and clinical symptoms are usually mild or absent, with recovery occurring within a few days. However, unvaccinated pregnant animals remain at high risk, especially if infection occurs during early to mid-gestation, which significantly increases the likelihood of severe congenital abnormalities, abortion, or stillbirth. Overall, the impact of SBV in endemic regions is relatively limited, but unvaccinated young breeding females remain vulnerable. Both vaccination and naturally acquired herd immunity play a crucial role in reducing new cases. In contrast, animals in non-endemic regions often lack immunity, and newly emerging outbreaks can result in a high rate of fetal malformations, potentially leading to severe economic losses to the livestock industry, including reduced reproductive efficiency and increased farming costs. Therefore, it is essential to implement targeted prevention and control strategies that are tailored to the specific conditions of both endemic and non-endemic regions. This approach will be more effective in controlling the spread of the disease and mitigating its impact.

## 4 Diagnosis for SBV

As SBV infection presents symptoms similar to those of other Bunyavirus infections, laboratory diagnostics are essential for accurate confirmation (Garigliany, 2012; Abutarbush et al., 2017). The primary diagnostic methods include virus isolation, molecular detection, and serological testing (**Table 1**).

**Table 1:** Diagnostic methods for SBV.

Type of diagnosis	Method	Sample Type	Application/Stage
Virus Isolation	Cell culture	Blood, placenta, brain tissue, vectors	Lab-based virology research
Molecular Tests	RT-qPCR	Serum, placenta, semen, vectors	Detection of SBV RNA during acute infection
	pan-Simbu RT-qPCR	Tissue, insects	Broad-range detection of multiple Simbu serogroup viruses, including SBV
	RT-PCR	Serum, placenta, semen, vectors	Confirmation and sequence analysis
	Digital PCR	Low-viral-load clinical samples	Confirming trace viral RNA post-infection
Serological Tests	ELISA	Serum, milk	Herd surveillance; historical exposure
	Virus Neutralization Test (VNT)	Serum	Confirmatory serology
Rapid Testing	Isothermal amplification (LAMP)	Blood, field samples	On-site or emergency testing
Genomic Tools	High-throughput sequencing (NGS)	Tissues, vectors	Viral evolution and transmission tracing

SBV can be isolated and cultured in a variety of insect and mammalian cell types, such as BHK-21, Vero, and KC cells (Hoffmann et al., 2012; Wernike et al., 2013b). However, due to the low viral load in most clinical samples, not all samples are successful in virus isolation, which limits its sensitivity.

Real-time fluorescence quantitative PCR (RT-qPCR) is the most commonly used molecular detection method (De Regge et al., 2013). It amplifies the S gene and L gene of the SBV genome to detect minute amounts of viral RNA with high sensitivity and specificity, making it especially suitable for early detection of acute infections and high-throughput testing. Samples from the placenta, serum, semen, and insect vectors can be used for RT-qPCR testing (Bilk et al., 2012; De Regge et al., 2013; Poel et al., 2014). However, since viremia is short-lived, the timing of detection is crucial. Some adult animals may have cleared the viral RNA by the time of testing, reducing the detection sensitivity. To address this limitation, digital PCR (dPCR) has been introduced, which increases sensitivity for low-load samples and reduces nonspecific interference, making it especially suitable for detecting trace amounts of RNA after virus clearance. Additionally, a broad-spectrum Simbu RT-qPCR detection method has been developed, which can be used to detect multiple Simbu serogroup viruses (Fischer et al., 2013; Golender et al., 2018; Camarão et al., 2019).

SBV-specific antibodies are typically produced 1–3 weeks after infection and can persist for several years. Therefore, serological testing is an effective means of diagnosing SBV infection. Virus neutralization tests (VNT), and enzyme-linked immunosorbent assays (ELISA) are the main serological tests (Loeffen et al., 2012; Beer et al., 2013). VNT is considered the “gold standard” due to its high specificity, but it is time-consuming and operationally complex, typically used only for confirmation (Loeffen et al., 2012). In contrast, ELISA is an efficient screening method that can detect antibodies not only in serum but also in milk, making it suitable for large-scale herd immunity screening and assessing infection history (Humphries and Burr, 2012; Bréard et al., 2013; Daly et al., 2015). However, in regions where other Simbu serogroup viruses co-circulate, the specificity of ELISA may be challenged (Blacksell et al., 1997). In a recent study conducted in Turkey’s Eastern Mediterranean region, the seroprevalence of SBV-specific antibodies detected by ELISA reached 29.11%, whereas only 3.17% of virological samples tested positive by RT-PCR, and no viral RNA was detected in vector samples. While this discrepancy may reflect the temporal gap between exposure and sampling, it may also suggest potential cross-reactivity or false positives inherent in serological assays (Dogan et al., 2022). In such settings, confirmatory testing by VNT becomes particularly essential to ensure diagnostic accuracy. This two-tiered diagnostic strategy has proven feasible in practice; for instance, a sero-epidemiological study in Spain (2006–2015) used ELISA for preliminary screening of wild ruminants, followed by VNT confirmation—demonstrating the reliability of this approach in both domestic and wildlife surveillance programs (García-Bocanegra et al., 2017).

To meet the demand for on-site rapid detection, various portable and rapid testing technologies have been developed in recent years. For example, isothermal amplification technologies, such as LAMP, have proven to perform effective in resource-limited environments due to their simplicity and low equipment requirements (Aebischer et al., 2014). Furthermore, novel detection platforms combining the CRISPR-Cas system have shown great potential, with sensitivity and specificity comparable to laboratory diagnostic technologies, significantly reducing detection times (Padmanaban and Ranganathan, 2022). The development and application of these rapid testing technologies provide crucial support for SBV screening in remote areas and emergency situations. Genome sequencing can track viral mutations and transmission chains, enhancing the epidemic monitoring system. As sequencing technology advances, the collection and analysis of genomic data have become essential tools for monitoring viral evolution and predicting transmission trends.

The epidemiological characteristics and transmission patterns of SBV vary significantly across regions, resulting in different requirements for diagnostic technologies. Diagnostic methods must be adjusted and optimized to the specific conditions of both endemic and non-endemic areas to ensure early detection, accurate assessment, and effective outbreak control.

### 4.1 Diagnosis in endemic regions

In European regions, where SBV is highly prevalent, the virus causes periodic outbreaks, typically recurring every 2–3 years (Cuéllar et al., 2018; Bayrou et al., 2022). During low prevalence periods, the virus can still be transmitted at low levels, making early detection crucial to prevent undiagnosed cases that could result in uncontrolled outbreaks. Therefore, Molecular diagnostic methods like RT-qPCR and digital PCR are prioritized during acute infection phases. They enable large-scale screening of samples, such as placenta and vector insects, within 24 hours. Additionally, serological monitoring of host immunity dynamics crucial for predicting the next wave of outbreaks. Most European countries have established comprehensive surveillance systems that regularly sample livestock populations and use RT-qPCR and serological tests to track virus transmission in real time (Roberts et al., 2014). To improve monitoring efficiency, some countries are exploring the use of machine learning-based epidemiological models to dynamically predict the virus spread and identify high-risk areas (Ali, 2024; Ekundayo, 2024; Liu et al., 2025).

The livestock industry in Europe is highly concentrated, especially in countries like Germany, the Netherlands, and France. Due to the large livestock populations, rapid testing and screening technologies are needed to reduce the risk of large-scale outbreaks. ELISA technology is widely used for large-scale serological screening due to its high throughput and cost-effectiveness, meeting this demand, while the VNT is used for high-precision confirmation testing of specific samples (Nurtop et al., 2018). Additionally, monitoring post-vaccination responses requires complementary tools to optimize the Differentiating Infected from Vaccinated Animals (DIVA) strategy, accurately distinguishing between naturally infected and vaccinated individuals, and assessing herd immunity levels and the potential for new outbreaks. To maintain high diagnostic standards, laboratories should regularly participate in inter-laboratory proficiency testing (Wernike and Beer, 2019).

In Asia and Africa, SBV outbreaks tend to be smaller in scale, but the potential risk of virus transmission should not be ignored (Zhai et al., 2018; Nadeem et al., 2024). The livestock industry in Asia is highly concentrated, particularly in countries like India and China, where dairy cattle and goat farming are widespread. While SBV outbreaks are currently rare, the active cross-border trade and livestock transport increase the risk of virus spread (Collins et al., 2019; Senf et al., 2020). In these regions, promoting portable diagnostic tools and establishing regional monitoring networks is highly recommended. In Africa, efforts to diagnose SBV face greater challenges due to limited laboratory facilities and diagnostic capabilities. Many remote areas lack basic laboratory infrastructure and specialized personnel, making a strong need for low-cost, easy-to-use field detection technologies. Diagnostics in these regions should focus on developing cost-effective tools, such as rapid test strip devices, which can provide results in just minutes, offering a significant advantage for rapid on-site screening. CRISPR-based diagnostic technologies, characterized by high sensitivity and specificity, hold great promise for improving field testing accuracy, particularly in rural livestock farming areas and during emergency outbreaks (Chertow, 2018; Ramachandran et al., 2020; Kaminski et al., 2021). One example is a CRISPR-Cas12 system combined with electric field control and microfluidics, which can detect SARS-CoV-2 RNA from raw samples in about 35 minutes (Ramachandran et al., 2020). This shows that CRISPR-based methods can be adapted quickly for different pathogens and could be a useful diagnostic tool for SBV. Additionally, the host range of SBV in African wildlife is still unclear, and the conditions for sample collection vary significantly among different species (Al-Busaidy et al., 1987; Mouchantat et al., 2015). Therefore, there is an urgent need to develop detection tools suitable for multiple sample types.

### 4.2 Diagnosis in non-endemic regions

Although SBV has not spread widely in non-endemic regions, the risk of virus introduction and spread has increased due to the rise in international trade and livestock transportation, especially through the importation of breeding livestock, frozen semen, and embryos, in which subclinically infected individuals may introduce the virus (Gibbens, 2012; Wernike et al., 2022). To address this challenge, a comprehensive prevention and control system must be established, focusing on a “blocking entry - early warning - rapid response” strategy. For imported livestock and reproductive products, RT-qPCR screening combined with serological ELISA/VNT tests to exclude subclinical infections and cross-reaction interference (Aebischer et al., 2014; Golender et al., 2018; Goto et al., 2023).

However, traditional detection methods have limitations in terms of screening speed and costs. To improve early diagnosis and monitoring efficiency, future efforts could focus on developing automated high-throughput screening technologies that enhance detection efficiency and reduce screening costs. Additionally, non-endemic regions should develop real-time monitoring systems based on data analysis and smart algorithms, establishing intelligent networks that integrate climate data (temperature, humidity), vector insect distribution models, and livestock immunization profiles (Robertson et al., 2010; Kapetas et al., 2025). AI algorithms can be used to predict high-risk areas. Drawing on cross-border monitoring experience from African swine fever, sharing virus gene sequences and epidemic dynamics with SBV-endemic countries is essential for timely updates to detection targets (Nadeem et al., 2024; Nie et al., 2024). A rapid response system should be implemented, with emergency protocols (e.g., isolation - re-testing - tracing) to ensure preliminary diagnosis is completed within 24 hours (Aebischer et al., 2014; Wernike and Beer, 2019). In border areas adjacent to endemic regions, pilot vector insect trapping and pathogen monitoring should be conducted to prevent the epidemic from infiltrating.

## 5 Vaccine development for SBV

The development of vaccines for SBV is a key strategy for controlling the virus. Currently, there are several types of vaccines under development, including inactivated vaccines, attenuated live vaccines, recombinant subunit vaccines, viral vector vaccines, and nucleic acid vaccines (**Table 2**) (Hechinger et al., 2014; Kraatz et al., 2015; Wernike et al., 2018b; Wernike and Beer, 2020). Among these, inactivated vaccines are the only vaccines currently approved for market use. Examples of approved vaccines include Bovilis SBV (MSD Animal Health), Zulvac SBV (Zoetis), and SBVvax (Merial). These vaccines function by chemically inactivating the viral particles, which triggers an immune response. Initially licensed in the United Kingdom and France in 2013, they subsequently received European Union-wide marketing authorization in May 2015. Inactivated vaccines have demonstrated high efficacy in preventing SBV infection, thereby significantly reducing the risk of fetal abnormalities and miscarriage in pregnant dams (Wernike et al., 2013d; Hechinger et al., 2014). A study showed that a single dose of an inactivated vaccine completely inhibited viral replication in all vaccinated sheep (5/5), as confirmed by competitive ELISA, microneutralization tests, and SBV-specific real-time RT-PCR (Hechinger et al., 2014). However, these vaccines come with some limitations, including high production costs, and the inability to differentiate between vaccinated and naturally infected animals.

**Table 2:** Vaccines developed against SBV.

Vaccine Types	Immunogen Design	Vector or Platform	Species Evaluated	Development Status
Inactivated vaccines	Inactivated virions	Chemical inactivation	Cattle, Sheep	Commercially licensed
Attenuated live vaccines	SBV ΔNSs/ΔNSm	Reverse genetics	Cattle, sheep	Preclinical evaluation
Recombinant subunit vaccines	Gc or Gc/Gn	Mammalian/Baculovirus	Cattle	Prototype vaccine
	N-protein	Bacterial		
Multimeric protein scaffold particle	Peptide epitopes displayed on LS-based nanoparticles	*A. aeolicus LS +* bacterial glue system	Cattle	Experimental
Viral vector vaccines	Gc head delivered by MVA, EHV-1	Recombinant viral vectors	Cattle	Experimental
Nucleic acid vaccines	SBV glycoproteins (Gc, Gn) and nucleoprotein (N, ± ubiquitination)	Mammalian expression vectors	IFNAR-/- mice	Experimental

Attenuated live vaccines are commonly developed through targeted deletion of viral genes, such as NSm or NSs, aiming to attenuate viral pathogenicity while preserving immunogenic properties (Bird et al., 2011; Kraatz et al., 2015). SBV mutants with deletions in the NSs and NSm genes were evaluated *in vitro*, in IFNAR^−^/^−^ mice, and in cattle. The double gene-deletion mutant demonstrated no detectable viral replication in cattle and conferred complete protection in all vaccinated animals following immunization. Importantly, this mutant also shows potential for DIVA capability (Kraatz et al., 2015; Varela et al., 2016). However, live vaccines may pose a risk of reversion to virulence, and further research and safety evaluations are needed before they can be widely used.

Recombinant subunit vaccines produce antigens through expression systems and induce immune responses with adjuvants, and are a key focus in SBV vaccine development. The envelope glycoproteins Gn and Gc mediate viral entry, with Gc identified as the main target of neutralizing antibodies. Sera from SBV-infected animals react strongly with the full-length Gc and its N-terminal domain, indicating that this region contains key neutralizing epitopes (Roman-Sosa et al., 2016). Several studies have evaluated the immunogenicity of Gc-based subunit vaccines. Kerstin et al. expressed the N-terminal domain of Gc using both prokaryotic and mammalian systems and assessed their protective efficacy in IFNAR^−^/^−^ mice and cattle (Wernike et al., 2017). Prokaryotically expressed forms generally failed to induce protective immunity, whereas mammalian-expressed proteins conferred partial protection in both models. A multivalent vaccine combining Gc domains from SBV and Akabane virus achieved full protection in animal models. Additionally, vaccinated animals lacked antibodies against the viral N-protein, allowing differentiation from natural infection, which supports a marker vaccine approach (Wernike et al., 2017). Subunit vaccines based on the Gc head–stalk construct have demonstrated strong protective efficacy, inducing sterilizing immunity in animal models. Compared to the head domain alone, the inclusion of the stalk enhances immune responses and protection. Moreover, the conserved structure of Gc across *Orthobunyaviruses* suggests potential for broad cross-protection (Roman-Sosa et al., 2016; Hellert et al., 2019). In addition to humoral responses, cellular immunity may also contribute to protection against SBV. Recent studies have explored the nucleoprotein (SBV-N) as an alternative immunogen. Bacterially expressed SBV-N, when combined with a veterinary-grade saponin adjuvant, reduced viremia and clinical signs in mice despite not inducing neutralizing antibodies, suggesting a role for T-cell–mediated immunity (Boshra et al., 2020). Further research showed that the C4 fragment of SBV-N elicited strong cellular responses and exhibited high sequence similarity with other Simbu viruses, supporting its potential as a broad-spectrum vaccine candidate (Guerra et al., 2023) While recombinant protein vaccines offer advantages in safety and DIVA compatibility, their immunogenicity depends on the stability and properties of the antigen, as well as the compatibility with adjuvants. Additionally, their relatively high production costs may limit their use in resource-limited regions.

Vaccines based on nanoparticles and protein structures are emerging as promising options in SBV vaccine development. Recently, the multimeric protein scaffold particle (MPSP) platform, derived from lumazine synthase (LS) of *Aquifex aeolicus*, has introduced a new strategy for vaccine design (Aebischer et al., 2021). This platform allows peptide epitopes to be presented via genetic fusion, while large antigens are conjugated to pre-assembled particles using bacterial “superglue”, enhancing the vaccine’s polyvalency and immunogenicity. In the study using SBV as a model, the MPSPs presented the key immunogens of the virus and showed strong protective effects in both mouse and cattle models. Compared to monomeric subunit vaccines, the multivalent antigens on the MPSPs significantly enhanced the immune response, with a single dose protecting 80% of mice from a lethal dose of SBV and inducing nearly sterile immunity in cattle (Aebischer et al., 2021).

Live viral vectors can serve as an effective delivery system for SBV Gc antigens, enabling efficient expression of the Gc protein *in vivo* and enhancing its immunogenicity. Kerstin et al. used modified Ankara vaccinia virus (MVA) and equine herpesvirus type 1 (EHV-1) attenuated strains as viral vectors, inserting the N-terminal of the SBV Gc glycoprotein to develop SBV live viral vector vaccines (Wernike et al., 2018b). The results showed that cattle vaccinated with the recombinant EHV-1 vector vaccine achieved 50% protection after challenge, while all cattle vaccinated with the recombinant MVA vector vaccine received complete immune protection, confirming that the MVA-delivered SBV Gc domain live viral vector vaccine exhibited higher immunogenicity (Wernike et al., 2018b). Due to the replication deficiency of MVA in mammals, it has a safety advantage over traditional vaccinia virus. Furthermore, since the recombinant MVA vector vaccine based on SBV Gc protein does not generate antibodies against the N protein in vaccinated animals, the use of N protein serological testing allows for DIVA compatibility of the vaccine.

Nucleic acid vaccines express viral antigens in host cells, activating both humoral and cellular immune responses, making them a promising vaccine development direction. DNA vaccines, which encode SBV Gc and N proteins, have demonstrated the ability to reduce viremia and provide partial protection, though they have only been tested in small animal models (Boshra et al., 2017). However, DNA vaccines face challenges such as low delivery efficiency, requiring optimization of delivery systems like electroporation or nanoparticle-based methods. mRNA vaccines have emerged a new direction for SBV vaccine research due to their short development cycles, high immunogenicity, and DIVA compatibility (Iavarone et al., 2017; Maruggi et al., 2019). The success of BNT162b2 and mRNA-1273 during the COVID-19 pandemic illustrates how quickly effective vaccines can be developed once the delivery platform is established (Baden et al., 2021; Khoury et al., 2021). Additionally, in recent years, mRNA has made significant progress in optimizing antigen design, improving immune efficacy, and enhancing delivery systems (Maruggi et al., 2019; Clemente et al., 2023; Mochida and Uchida, 2024). It is believed that mRNA vaccines for SBV will play an important role in disease prevention and control in the future.

Although significant progress has been made in the research of various types of SBV vaccines, vaccine development and vaccination strategies must fully consider factors such as the intensity of outbreaks, geographic conditions, economic constraints, and technical accessibility. These strategies should be tailored to the specific characteristics of the epidemic in different regions to ensure maximum effectiveness and feasibility of vaccine implementation.

### 5.1 Vaccination strategies in endemic regions

In Europe, the main endemic region for SBV, vaccination strategies focus on establishing and maintaining herd immunity to prevent viral infections that cause fetal deformities and miscarriages (Hoffmann et al., 2012; Veldhuis et al., 2017). Studies show a high seroprevalence of SBV in these regions, with many adult animals acquiring immunity through natural infection. However, first-time pregnant dams remain at significant risk of infection. Additionally, due to changes in livestock populations, older animals with natural antibodies are gradually replaced by younger, susceptible animals, leading to a decline in herd immunity levels and promoting virus re-circulation (Bessell et al., 2014; Wernike et al., 2018a). Therefore, regular vaccination, particularly targeting susceptible young dams, may be an effective control strategy.

Inactivated vaccines have been widely used and, when combined with seasonal monitoring, help reduce the risk of outbreaks. Studies show that a single dose of the vaccine can induce sufficient antibody levels within two weeks (Wernike et al., 2013d). Vaccination is recommended to be completed prior to mating or during early gestation to confer protective immunity throughout pregnancy and to ensure that maternal antibody levels reach a protective threshold during the critical period of fetal susceptibility (Martinelle et al., 2015; König et al., 2019). Due to the uncertainty of SBV transmission, some regions may use emergency vaccination after an outbreak. However, it should be noted that emergency vaccination may be less effective in pregnant animals (Græsbøll et al., 2014).

The demand for new vaccines in European endemic areas is gradually increasing, especially those that support DIVA diagnostic strategies. These vaccines not only optimize epidemic dynamic monitoring but also ensure smooth health certification in livestock trade, thus preventing economic losses caused by trade restrictions. Additionally, by integrating epidemiological modeling technology, predictive tools can be used to dynamically adjust the vaccination timing and coverage, enabling more precise control. Vaccination in endemic regions should be integrated with molecular and serological diagnostic techniques to create a dual defense system for both immunity and monitoring (Wernike and Beer, 2020).

SBV prevalence in Asia and Africa is lower than in Europe, but cross-border trade and natural transmission risks are increasingly evident. Vaccination strategies should focus on key breeding livestock populations, combined with vector control measures to reduce the risk of virus transmission. Studies show that genetically engineered attenuated live vaccines can provide long-lasting immunity with a single dose and reduce cold chain transportation requirements. Moreover, research is exploring oral or spray vaccine delivery methods to lower vaccination costs and improve herd immunity coverage. In regions with dense wild animal populations or complex breeding environments in Africa, vaccine distribution should be adapted to local ecological conditions to ensure coverage of a sufficiently wide host population (Songane, 2018).

In summary, SBV vaccination strategies in endemic regions extend beyond vaccination alone, encompassing rigorous surveillance, ongoing vaccine development, and informed policy implementation to effectively mitigate the impact of the virus on the livestock industry.

### 5.2 Vaccination strategies in non-endemic regions

In non-endemic areas such as North America and Australia, vaccination strategies should focus on preventing virus introduction and enabling rapid response in the event of an outbreak. The main risks arise from international trade and cross-border livestock transportation, especially through the import of breeding animals, frozen semen, and embryos, through which the virus may enter local herds via subclinical infections. Therefore, the core task of vaccination is not large-scale distribution, but rather the establishment of emergency immunization reserves. Vaccine stockpiles should be set up in major livestock trade hubs and breeding centers to ensure rapid deployment in case of an outbreak. Since inactivated vaccines cannot differentiate between infected and immune animals, and attenuated live vaccines pose risks of reversion to virulence and environmental leakage, non-endemic areas are more likely to choose new vaccines that support DIVA strategies, such as subunit vaccines, protein scaffold-based vaccines, and nucleic acid vaccines. Particularly, protein scaffold-based vaccines are promising for providing rapid protection, reducing the need for frequent vaccinations, and should be prioritized for emergency reserves (Aebischer et al., 2021). Furthermore, mRNA vaccines, with their ability to induce a comprehensive immune response and their rapid production advantages, will also be suitable for emergency immunization once successfully developed (Maruggi et al., 2019). Notably, emergency vaccination should be integrated with cross-border quarantine systems. Once an imported case is detected, emergency vaccination should be carried out for susceptible animals around the outbreak point to ensure immunity coverage. To address potential viral mutations, vaccine stockpiles should be regularly evaluated for efficacy and updated according to the standards of the World Organisation for Animal Health (WOAH). In summary, vaccination strategies in non-endemic regions should focus on establishing emergency immunization reserves and rapid response mechanisms, prioritizing the use of innovative vaccines that support DIVA strategies to manage the risk of virus introduction and improve immunity coverage. By combining cross-border quarantine and dynamically adjusted vaccine reserves, the risk of virus transmission can be effectively reduced, ensuring the sustainability of control measures.

## 6 Vector control strategies for SBV

Effective control of vector-borne viruses such as SBV requires more than just epidemiological surveillance and vaccination (Gubbins et al., 2014; Sumner et al., 2017; Achee et al., 2019). Central to this is the management of *Culicoides* midges—the primary vectors responsible for transmission. By integrating chemical control, biological control, environmental and farm interventions, and climate monitoring strategies, a comprehensive and multidimensional vector control system can be established, facilitating early warning and rapid response to SBV outbreaks.

Key chemical strategies include using pyrethroid-based adulticides during peak midge activity seasons via ground spraying, indoor residual spraying, or insecticide-treated materials in livestock housing (Gubler, 2005). Larvicidal agents such as insect growth regulators (IGRs)—including pyriproxyfen and methoprene—are used to disrupt the development of immature stages at breeding sites (Baldacchino et al., 2015). While these interventions can rapidly reduce vector abundance, their sustained or indiscriminate use poses challenges such as insecticide resistance, ecological toxicity, and negative impacts on non-target species (Wernike et al., 2013b; Snyder et al., 2016; Matsuo, 2019).

Biological control strategies have emerged as sustainable alternatives (Huang et al., 2017). This approach employs natural agents—such as entomopathogenic fungi, bacterial larvicides, and aquatic predators—to reduce vector populations or impair their transmission capacity. Fungi like *Beauveria bassiana* and *Metarhizium anisopliae* have demonstrated efficacy against various arthropods, though their performance is sensitive to environmental conditions including temperature, humidity, and formulation parameters (Ansari et al., 2011; Fernandes et al., 2012). Bacterial larvicides, such as *Bacillus thuringiensis* var. *israelensis* and *Lysinibacillus* sp*haericus*, produce toxins specifically targeting mosquito larvae, and their combined application can help mitigate resistance development (Silva-Filha et al., 2021).

Environmental and farm interventions are equally crucial. Reducing standing water, improving drainage, and optimizing barn structures can limit *Culicoides* breeding sites—especially in intensive farming systems (Lievaart-Peterson et al., 2015b; Kohara et al., 2018). Small-scale farms can further minimize vector-host contact using physical barriers like insect-proof screens and nets. Adjusting livestock reproduction schedules to avoid peak vector activity, along with evening stabling and rotational grazing, are practical risk-reduction tactics. International trade and transport pose significant risks for vector and pathogen spread, requiring stringent inspection, disinfection protocols, and vector surveillance at ports, airports, and border crossings to mitigate introductions via cargo, vehicles, and animal movements.

Climate factors—particularly rising temperatures, shifting precipitation patterns, and wind dynamics—play a pivotal role in SBV transmission (De Regge, 2017). Elevated temperatures can extend the seasonal activity of *Culicoides* midges, while altered precipitation reshapes breeding habitat distribution (Haider et al., 2018). Wind influences midge flight and host-seeking behavior, thus modulating viral spread (Mellor et al., 2000). These drivers collectively determine the spatiotemporal dynamics of SBV, especially in temperate zones where vectors are climate-sensitive. Establishing robust meteorological monitoring systems and integrating climate, vector ecology, and host data into predictive models is essential. The incorporation of real-time satellite data on land surface temperature, vegetation indices, and precipitation enhances early warning accuracy and guides timely control measures (Ceccato et al., 2018; Pley et al., 2021). Additionally, wind conditions should be incorporated into risk assessments to guide livestock movement and housing strategies aimed at reducing vector-host contact.

A One Health approach that fosters collaboration across veterinary, entomological, environmental, and public health disciplines is fundamental to devising effective, climate-resilient, and region-specific SBV control strategies (Socha et al., 2022). Tailoring interventions based on local climate, vector presence, livestock production systems, and trade patterns enhances prevention efficacy and sustainability. While many control measures can be broadly applied, their successful implementation must reflect the distinct epidemiological contexts of endemic versus virus introduction-risk areas.

### 6.1 Vector control in endemic regions

In SBV-endemic areas with established virus circulation, vector control aims to suppress transmission cycles and reduce disease impact on livestock health and productivity. Control strategies emphasize sustained application of chemical insecticides timed to vector seasonal peaks, often supported by biological control, which helps limit insecticide reliance and resistance. Environmental management is intensive, focusing on eliminating vector breeding habitats within and around farms, combined with physical barriers to minimize vector-host interactions. Livestock management—such as adjusting reproduction to avoid peak vector seasons and housing animals during high-risk periods—is a key component of risk mitigation. Biosecurity within endemic zones centers on preventing virus reintroduction and spread among herds, involving quarantine of new stock and disinfection protocols (Pley et al., 2021). Furthermore, evidence indicates that climate change has extended the active vector season and increased the number of transmission cycles. To address these emerging challenges in endemic regions, monitoring systems should integrate high-resolution satellite data with local vector surveillance to produce real-time SBV risk maps (Fairbanks et al., 2024). Climate-driven surveillance and modeling inform adaptive management strategies, ensuring timely and effective deployment of control measures tailored to shifting vector population dynamics.

### 6.2 Vector control in non-endemic regions

Non-endemic regions prioritize prevention of virus and vector introduction (Roberts et al., 2014). With no active circulation, control efforts focus on robust border biosecurity, including quarantine and inspection of animals and goods, disinfection of transport vehicles, and surveillance of vectors at entry points such as airports and seaports. Environmental and livestock management aim at reducing potential vector habitats in areas surrounding livestock operations, particularly near international trade hubs. Physical barriers and husbandry practices serve as preventive buffers, limiting any inadvertent contact between vectors and susceptible animals (Narladkar, 2018). Surveillance in non-endemic areas focuses on early detection and rapid response to vector incursions or virus introductions, guided by climate and ecological modeling to anticipate and mitigate invasion risks before local transmission can be established. Predictive models based on satellite climatic data can help identify areas that may become suitable for vector introduction due to climate shifts. Surveillance should prioritize these high-risk zones with sentinel herd monitoring, vector trapping, and environmental assessments.

## 7 Conclusion and future perspectives

SBV is an orthobunyavirus transmitted by *Culicoides* midges, with its epidemiological characteristics influenced by multiple factors, including climate change, vector ecology, livestock management, and international trade (Walter and Barr, 2011; Hoffmann et al., 2013; Wernike et al., 2014). This paper systematically reviews pathogen biology, epidemiology, detection methods, vaccine development, and vector control strategies for SBV. Based on the differing needs of endemic and non-endemic regions, differentiated and comprehensive response strategies are proposed (**Table 3**). In endemic regions, SBV exhibits periodic outbreaks, requiring a comprehensive control approach that includes vaccination, pathogen and serological monitoring, and vector management, in order to minimize economic losses in the livestock industry. In contrast, non-endemic regions should focus on managing border and import risks, including monitoring for pathogens and vectors, and strengthening emergency immunization reserves to prevent cross-border transmission of the virus and reduce the risk of outbreaks.

**Table 3:** SBV control strategies in endemic vs non-endemic areas.

Category	Endemic Areas	Non-Endemic Areas
Diagnostic Strategies	➢ Routine RT-qPCR for infection monitoring ➢ Regular ELISA screening + VNT confirmation ➢ Serological surveillance of herd immunity ➢ Use of AI for outbreak prediction	➢ Entry screening for imported animals (RT-qPCR + ELISA) ➢ Emergency diagnostics during suspected outbreaks Cross-border data sharing & AI-based early warning
Vaccination Strategies	➢ Periodic vaccination to maintain herd immunity ➢ Focus on naïve or pregnant animals ➢ Model-driven timing optimization ➢ inactivated and attenuated live vaccines	➢ Prioritize DIVA-compatible vaccines (subunit vaccines, protein scaffold-based vaccines, and nucleic acid vaccines) ➢ Emergency vaccine stockpiling ➢ Rapid deployment during outbreaks
Vector Control Strategies	➢ Continuous vector suppression (chemical + biological) ➢ Habitat elimination and stabling during peak vector seasons ➢ Isolate new stock and disinfect facilities	➢ Border quarantine and vector monitoring at ports ➢ Environmental control near livestock trade zones ➢ Rapid vector response upon detection

Although available control technologies have played a significant role in epidemic monitoring in both endemic and non-endemic regions, they still face several key challenges. Currently, RT-qPCR, dPCR, and ELISA are widely used for virus monitoring, while rapid detection technologies such as LAMP also show promising applications (Aebischer et al., 2014; Daly et al., 2015; Nurtop et al., 2018; Goto et al., 2023). However, these methods still have limitations in terms of specificity, sensitivity, and applicability, particularly in resource-limited areas. There is a need to accelerate the development of faster, simpler, and field-appropriate detection technologies to improve monitoring efficiency and accuracy. In vaccine development, the existing inactivated vaccines remain the only approved type, but they are unable to differentiate between vaccinated and naturally infected animals (DIVA incompatibility) (Endalew et al., 2019). Researchers are exploring new vaccine types such as recombinant protein, vector, DNA/mRNA, and VLP vaccines to address these issues. The goal of developing these novel vaccines is not only to enhance immune efficacy but also to ensure biosafety and support disease control and eradication in endemic regions. Furthermore, vaccine development must also take into account regional resource constraints and production capacities, particularly in resource-poor areas, where there is a need to develop low-cost and heat-stable vaccine technologies to ensure broad applicability.

Given the challenges posed by the cross-border transmission of SBV, global cooperation is crucial for establishing a systematic SBV control strategy (Ma et al., 2022). To develop precise control measures, strengthening international data sharing, technology transfer, and cross-national epidemiological research is essential (Chokshi et al., 2006; Lang, 2011). Additionally, establishing a global animal disease monitoring system, optimizing vaccine stockpiling mechanisms, and enhancing cross-border surveillance networks can effectively slow the rapid spread of outbreaks. As climate change increasingly affects virus transmission, integrating real-time meteorological monitoring, climate modeling, and remote sensing technologies will not only improve prediction capabilities for vector-borne viruses but also facilitate more precise cross-border control measures (Davis et al., 2017; Merkord et al., 2017; Wimberly et al., 2022). By establishing a multi-layered global cooperation framework, a solid foundation can be laid for the long-term control of SBV and other emerging viruses, ensuring the sustainable development of livestock industries.
